# A Series of Unfortunate Events: Prinzmetal Angina Culminating in Transmural Infarction in the Setting of Acute Gastrointestinal Hemorrhage

**DOI:** 10.1155/2013/641348

**Published:** 2013-04-23

**Authors:** Michael Ruisi, Phillip Ruisi, Hugo Rosero, Paul Schweitzer

**Affiliations:** ^1^Beth Israel Medical Center, Albert Einstein College of Medicine, New York, NY, USA; ^2^Rhode Island Hospital, Providence, RI, USA

## Abstract

Prinzmetal angina or vasospastic angina is a clinical phenomenon that is often transient and self-resolving. Clinically it is associated with ST elevations on the electrocardiogram, and initially it may be difficult to differentiate from an acute myocardial infarction. The vasospasm induced in this setting occurs in normal or mildly to moderately diseased vessels and can be triggered by a number of etiologies including smoking, changes in autonomic activity, or drug ingestion. While the ischemia induced is usually transient, myocardial infarction and life-threatening arrhythmias can occur in 25% of cases. We present the case of a 65-year-old female where repetitive intermittent coronary vasospasm culminated in transmural infarction in the setting of gastrointestinal bleeding. This case highlights the mortality associated with prinzmetal angina and the importance of recognizing the underlying etiology.

## 1. Introduction

Coronary vasospastic angina also known as Prinzmetal angina is a discrete clinical entity characterized as episodic angina pectoris in association with ST-segment elevations on electrocardiogram in the absence of high-grade coronary artery stenosis. These episodes usually occur at rest and often between the midnight and early morning hours [1]. The etiology of Prinzmetal angina is believed to be focal spasm of the smooth muscle layer of the arterial wall. These spasms occur in normal or mildly diseased vessels in the absence of any preceding increase in myocardial demand [1–4]. Transient ischemia is responsible for the anginal symptoms, while myocardial infarction can develop in a percentage of patients. Myocardial infarction and life-threatening arrhythmias are believed to occur in 25% of untreated patients with Prinzmetal angina [5]. The major risk factor contributing to vasospastic angina is thought to be an active smoking history [6]. Other possible triggers include changes in autonomic activity, the use of ephedrine-based products, cocaine ingestion guide wire or balloon dilatation, and magnesium deficiency [7–10]. It is usually diagnosed before 50 years of age with a higher prevalence in females and in the Japanese population [11]. Diagnosis may be challenging and includes ambulatory monitoring for ST segment elevations as well as exercise stress testing. In some scenarios intracoronary provocative tests can be performed in the catheterization lab to secure the diagnosis. Acetylcholine and ergonovine are the most widely used drugs for these tests. Treatment modalities have focused on calcium channel blockers for prevention and nitroglycerin in the acute setting. For severe vasospastic angina, percutaneous intervention with placement of a stent in the effected vessel has shown success [12–17]. We present the case of severe coronary vasospasm in a 65-year-old female in the setting of acute gastrointestinal hemorrhage.

## 2. Case Presentation

**Figure 1:**
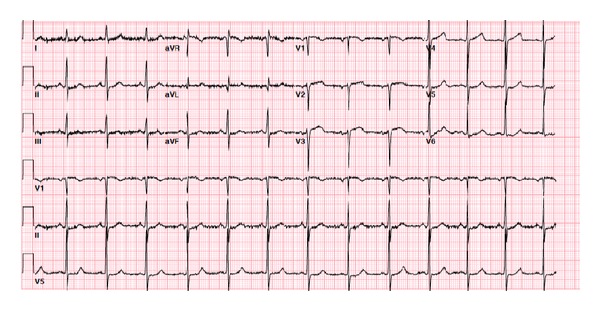


A 65-year-old female presented to our institution with a chief complaint of syncope and chest pain. She had a past medical history of untreated hypertension and recently diagnosed peptic disease with noncompliance to proton pump therapy. Additionally she reported a 30-pack year tobacco history with continued use. In the afternoon of her admission, she was walking from her house to her mailbox, when she developed sudden onset of chest pressure and tightness. She subsequently lost consciousness and was awoken by emergency medical personnel. The duration of loss of consciousness is unclear, and there was no rhythm strip reported by the EMS staff. She denied any prodromal symptoms of lightheadedness, nausea, and palpitations prior to the syncopal event. Furthermore, there were no signs or evidence of a post-ictal state. She was immediately transferred to our facilities emergency room where she was fully examined and reported no symptoms on initial triage. Her presenting electrocardiogram was largely nonspecific with a normal sinus rhythm and no significant ST or T wave abnormalities suggestive of an ischemic process (ECG #1) (Figure 1). 

**Figure 2:**
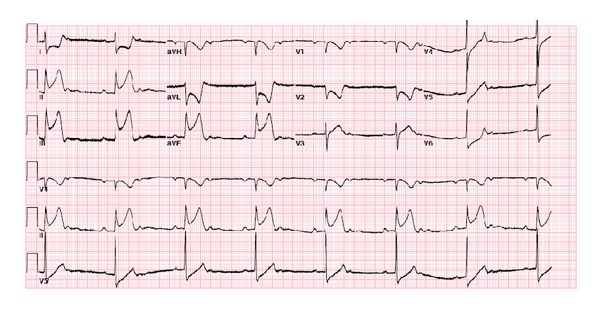


 An initial troponin I level of 0.2 prompted treatment for a non-ST elevation myocardial infarction. She was given aspirin 325 mg, plavix 600 mg, and an appropriate weight-based dose of lovenox. She continued to be symptom free for three hours while being in our emergency room. Approximately three and half hours after her arrival to our institution, she developed sudden onset of chest pressure and pain, with a rapid deterioration in her clinical status. She was bradycardic with heart rate 30–35, and blood pressure readings of 60s systolic. She appeared pale, diaphoretic, and in severe distress. A repeat ECG at the time was significant for 2 : 1 sinus bradycardia with a prolonged PR interval, as well as significant ST elevations (5-6 mm) in the inferior leads with ST depressions (2-3 mm) anteriorly (ECG #2) (Figure 2). 

**Figure 3:**
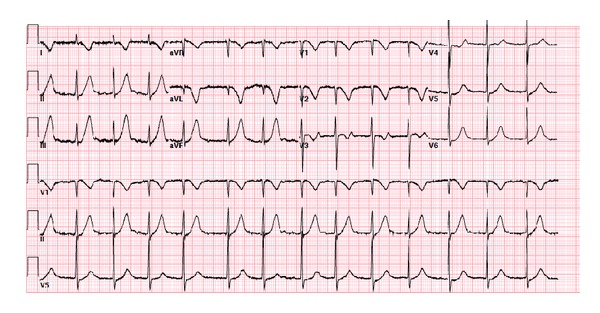


This clinical status persisted for approximately ten minutes when suddenly the heart rate increased to 90, and systolic blood pressure increased to 130s. Her symptoms had completely resolved and a repeat ECG demonstrated complete resolutions of the ST segment elevations (ECG#3) (Figure 3). 

 At this time cardiology was notified, and the patient was taken immediately for coronary angiography under the presumption for an acute coronary syndrome. In transport to the catheterization lab, the patient developed profuse melanotic stools mixed with evidence of bright red blood. 

In the catheterization lab, her radial artery was canalized with a 6 French sheath for coronary access. Multiple views of a diagnostic coronary angiogram failed to reveal any culprit lesion and all three major vessel beds demonstrated nonobstructive disease. The angiographic findings in conjunction with the clinical course suggested severe coronary vasospasm as the etiology for the patient's condition. Intra-coronary provocative testing was not undertaken given the risk associated with the current acute situation. The patient was subsequently transferred to coronary care unit overnight for closer monitoring. There was no significant elevation in cardiac biomarkers. Throughout the night she developed multiple bouts of hematochezia with a decline in hemoglobin level to 5.4 from the initial 10.6 level on admission. The patient was treated for hypovolemic shock secondary to gastrointestinal hemorrhage and urgent endoscopy was performed revealing multiple duodenal ulcers that were cauterized. The following morning the patient developed another episode of coronary vasospasm with the ECG changes identical to the initial episode. Despite endoscopic therapy, the gastrointestinal hemorrhage did not cease. The vasospasms continued to occur intermittently over a 48-hour period with a frequency of 10–15 minutes culminating in complete heart block and hemodynamic instability. Nitroglycerin was attempted temporarily for relief of the vasospasms, but the intermittent hypotension with the vasospasms precluded its clinical effectiveness. Placement of a transvenous pacer wire was attempted unsuccessfully. Unfortunately the patient expired approximately 72 hours after her arrival to our institution. Just prior to her cardiac arrest, a troponin I level of 140 was noted confirming our suspicion that the coronary spasms had lead to myocardial infarction. 

## 3. Discussion

In the aforementioned case described above, intense and frequent episodes of coronary vasospasm contributed to worsening cardiac function and eventual transmural infarction. The gastrointestinal bleeding was incessant throughout the hospital course and likely the inciting event for the coronary vasospasms. In general, patients with variant angina can have significant morbidity, but mortality however tends to be low in most cases. In a long-term follow-up study by Bory et al. in 1996, 277 successive patients with diagnosed Prinzmetal angina were followed for a median time of 89 months. At the end of the study, 6.5% of the patient developed myocardial infarction, and 3.6% died from cardiac causes [18]. This case is unique in that it highlights the potential severity and unexpected mortality that can be associated with Prinzmetal angina. Unfortunately in this particular case, the presumed inciting event was not remedied successfully. This case underscores the importance of recognizing the severity of Prinzmetal angina and the potential need for reversing the underlying etiology.

## References

[1] Prinzmetal M, Kennamer R, Merliss R, Wada T, Bor N (1959). Angina pectoris I. A variant form of angina pectoris: preliminary report. *The American Journal of Medicine*.

[2] Kaski JC, Crea F, Meran D (1986). Local coronary supersensitivity to diverse vasoconstrictive stimuli in patients with variant angina. *Circulation*.

[3] Kaski JC, Maseri A, Vejar M, Crea F, Hackett D, Halson P (1989). Spontaneous coronary artery spasm in variant angina is caused by a local hyperreactivity to a generalized constrictor stimulus. *Journal of the American College of Cardiology*.

[4] Prinzmetal M, Ekmekci A, Kennamer R, Kwoczynski JK, Shubin H, Toyoshima H (1960). Variantform of angina pectoris, previously undelineated syndrome. *Journal of the American Medical Association*.

[5] Okumura K, Yasue H, Matsuyama K (1996). Diffuse disorder of coronary artery vasomotility in patients with coronary spastic angina: hyperreactivity to the constrictor effects of acetylcholine and the dilator effects of nitroglycerin. *Journal of the American College of Cardiology*.

[6] Takaoka K, Yoshimura M, Ogawa H (2000). Comparison of the risk factors for coronary artery spasm with those for organic stenosis in a Japanese population: role of cigarette smoking. *International Journal of Cardiology*.

[7] Lanza GA, Pedrotti P, Pasceri V, Lucente M, Crea F, Maseri A (1996). Autonomic changes associated with spontaneous coronary spasm in patients with variant angina. *Journal of the American College of Cardiology*.

[8] Stern S, DeLuna AB (2009). Coronary artery spasm: a 2009 update. *Circulation*.

[9] Forman MB, Blass M, Jackson EK (2011). Variant angina in the setting of food-borne botulism. *Clinical Infectious Diseases*.

[10] Satake K, Lee JD, Shimizu H, Ueda T, Nakamura T (1996). Relation between severity of magnesium deficiency and frequency of anginal attacks in men with variant angina. *Journal of the American College of Cardiology*.

[11] Braunwald E (2011). *Heart Disease: A Textbook of Cardiovascular Medicine*.

[12] Araki H, Koiwaya Y, Nakagaki O, Nakamura M (1983). Diurnal distribution of ST-segment elevation and related arrhythmias in patients with variant angina: a study by ambulatory ECG monitoring. *Circulation*.

[13] Lahiri A, Subramanian B, Millar-Craig M (1980). Exercise-induced S-T segment elevation in variant angina. *American Journal of Cardiology*.

[14] Song JK, Park SW, Kang DH (2000). Safety and clinical impact of ergonovine stress echocardiography for diagnosis of coronary vasospasm. *Journal of the American College of Cardiology*.

[15] Hamilton KK, Pepine CJ (2000). A renaissance of provocative testing for coronary spasm?. *Journal of the American College of Cardiology*.

[16] Yamada T, Okamoto M, Sueda T, Hashimoto M, Matsuura H, Kajiyama G (1998). Ergonovine-lnduced alterations in coronary flow velocity preceding onset of occlusive spasm in patients without significant coronary artery stenoses. *American Journal of Cardiology*.

[17] Pepine CJ, Feldman RL, Conti CR (1982). Action of intracoronary nitroglycerin in refractory coronary artery spasm. *Circulation*.

[18] Bory M, Pierron F, Panagides D, Bonnet JL, Yvorra S, Desfossez L (1996). Coronary artery spasm in patients with normal or near normal coronary arteries. Long-term follow-up of 277 patients. *European Heart Journal*.

